# The great awakening: A 15-year bibliometric analysis of the global surge in sleep research

**DOI:** 10.1007/s11325-026-03733-9

**Published:** 2026-06-10

**Authors:** Isabela A. Ishikura, Allan Saj Porcacchia, Sergio Tufik, Monica L. Andersen

**Affiliations:** 1 Departamento de Psicobiologia, Universidade Federal de São Paulo (UNIFESP), São Paulo, SP Brazil; 2 Instituto do Sono, Associação Fundo de Incentivo à Pesquisa (AFIP), São Paulo, SP Brazil

**Keywords:** Sleep, Insomnia, Obstructive sleep apnea, Sleep quality, Interdisciplinary health, Bibliometric analysis

## Abstract

**Purposes:**

To conduct a longitudinal bibliometric analysis of sleep-related research output across 6 major health disciplines and to identify the specific sleep variables most explored within these fields.

**Methods:**

Using the Journal Citation Reports (JCR), the top 10 high-impact journals from 6 categories were evaluated: Clinical Psychology, Nutrition & Dietetics, Pediatrics, Dentistry, Geriatrics & Gerontology, and Sports Sciences. Publication trends for the term “sleep” were analyzed in Web of Science Core Collection (WoS) database across 3 5-year timeframes: 2010–2014 (T1), 2015–2019 (T2), and 2020–2024 (T3). Total publication volume of each journal, relative percentage growth in term “sleep”, and prevalence of specific sleep-related descriptors (e.g., insomnia, obstructive sleep apnea - OSA, sleep quality) were quantified.

**Results:**

Sleep-related research accounted for 0.4% to 2.2% of the total scientific output across the analyzed categories. While total scientific publication volume increased across all fields over the 15-year period, the growth of sleep-related research outpaced general journal expansion by more than 1.5-fold in 4 of the 6 categories. The surge between T2 and T3 was most pronounced in Sport Sciences (140.6%) and Geriatrics (49.4%). Overall, the total relative increase from T1 to T3 was most substantial in Sport Sciences (305.3%) and Clinical Psychology (95.5%), followed by Dentistry (60.0%) and Geriatrics (51.2%). Insomnia, OSA and Sleep Quality were the most frequent descriptors across disciplines.

**Conclusions:**

Our findings reveal that diverse scientific disciplines are increasingly incorporating sleep as a key research variable, as evidenced by the significant growth in sleep-related publications within their specialized journals. This trend underscores a growing recognition of sleep’s fundamental role in physiological mechanisms and its crucial influence on health. The broader dissemination of sleep science across these distinct fields enables researchers to develop specialized interventions aimed at reducing pathologies and enhancing quality of life through domain-specific practices.

**Clinical Trial Registration:**

not applicable for this type of study.

**Supplementary Information:**

The online version contains supplementary material available at 10.1007/s11325-026-03733-9.

## Introduction

Sleep, a physiological state occupying approximately one-third of the human lifespan, remains one of the most complex frontiers in biological research. Characterized by reduced environmental reactivity and a suspension of conscious awareness, the scientific understanding of sleep underwent a paradigm shift in 1953 with Aserinsky and Kleitman’s discovery of rapid eye movement (REM) [1]. This landmark identification of cyclical, active brain states during sleep catalyzed decades of global inquiry, transforming sleep from a perceived state of rest into a dynamic field of neurobiological study.

The regulatory mechanisms of sleep are now well-established, centered on the hypothalamic control of sleep onset and the master circadian pacemaking of the suprachiasmatic nuclei [1]. However, contemporary research has moved beyond simple neuroanatomy to reveal that sleep is a fundamental homeostatic regulator of systemic health. Recent evidence has elucidated its critical roles in immunomodulation [2], endocrine signaling [3], and the maintenance of neurocognitive integrity [4].

Despite its biological necessity, sleep quality has been systematically devalued in modern society. Driven by an ethos of relentless productivity and the ubiquity of digital stimulation, individuals increasingly truncate sleep to accommodate extended work hours and social demands. This chronic sleep deprivation is a major contributor to the surge in “lifestyle pathologies,” including obesity, metabolic syndrome, and cardiovascular disease [5]. The rising prevalence of sleep-disordered breathing, primarily obstructive sleep apnea (OSA), linked to the obesity epidemic, and stress-induced insomnia has created a public health crisis that transcends traditional medical boundaries [4, 6].

Sleep is now recognized as one of the 3 foundational pillars of health, alongside physical activity and nutrition [7]. These components are intrinsically linked, creating a synergistic effect on overall well-being. This realization has sparked a “translational revolution,” where sleep science is no longer confined to neurology or psychiatry. Instead, its relevance has permeated diverse healthcare domains, from the biomechanics of sports and the metabolic pathways of nutrition to the structural assessments in dentistry and the developmental milestones in pediatrics.

Bibliometric studies focusing on sleep have demonstrated the progression of sleep science over the decades, with a 4-fold increase in the number of sleep publications between 1974 and 2004, although the general publication reached half of this increase [8]. In the same direction, bibliometric research of OSA-related articles reported an increase of 582 number of articles in this topic during 1991 and 2012, with USA leading the rank of publication in the field [9].

Due to its diverse and complex consequences in body physiology, sleep has become recognized as an important element for several disciplines of health, *but which of them have been more concerned about sleep? Does the growth in sleep-related publications prevail in different disciplines?* Understanding these trends allows specialists to identify emerging clinical priorities, ultimately fostering a more informed approach to patient care and interdisciplinary referrals.

This study aimed to investigate the longitudinal growth of sleep-related publications across 6 distinct health science categories—Psychology, Sports, Nutrition, Geriatrics, Pediatrics, and Dentistry—over a 15-year period (2010–2024). By analyzing publication trends within top-tier journals in each field, we seek to map the expanding landscape of sleep science and identify the specific sleep dimensions gaining prominence across the health sciences.

## Methods

### Study design and journal selection

This longitudinal bibliometric study, conducted in April 2026, analyzed publication trends across 6 health-related categories. Journals were selected based on the Journal Citation Reports (JCR). For each of the following categories, the top 10 journals by impact factor were evaluated: Clinical Psychology; Nutrition & Dietetics; Pediatrics; Dentistry, Oral Surgery & Medicine; Geriatrics & Gerontology; and Sport Sciences. A complete list of the 60 selected journals and their respective impact factors is provided in **Supp.** Table 1. For brevity, categories are henceforth referred to by their primary descriptor (e.g., “Nutrition” for Nutrition & Dietetics). The inclusion criteria were the top 10 journals of JCR. No exclusion criteria were considered in this study.

The JCR was intentionally selected over broader bibliographic databases to prioritize disciplinary precision and focus on high-impact research trends. The selection of these 6 specific JCR categories was a deliberate methodological choice designed to investigate the interdisciplinary expansion of sleep science outside its traditional medical silos (such as Neurology or Pulmonology). Pediatrics and Psychology were selected as established pillars where sleep is critical for neurodevelopment and mental health; Geriatrics and Nutrition were included to test the hypothesis of sleep as a metabolic and longevity variable; Dentistry and Sports Sciences represent specialized fields where sleep-related interventions (e.g., intraoral appliances, recovery performance) have gained significant clinical momentum.

### Data collection and search strategy

The data collection was performed using the Web of Science Core Collection (WoS) database with 3 distinct phases of search strategy. Results were filtered into 3 distinct 5-year timeframes:

#### T1

 January 1, 2010, to December 31, 2014.

#### T2

January 1, 2015, to December 31, 2019.

#### T3

January 1, 2020, to December 31, 2024.

Specifics queries were inserted in Advanced Search Query of the WoS to eliminate the variability inherent in individual journal platforms. To ensure thematic relevance and avoid false positives, the search was restricted to the title, abstract and keywords fields. This standardized approach allows for full reproducibility of the thematic growth trends identified in each category. No document type filters were applied to ensure that the total denominator reflected the full editorial activity of each source.

#### Phase I

Longitudinal publication trends: to investigate the number of sleep-related publications using the term “sleep” in each journal. The query contained the following terms, that were replaced according to the specific period and journal. Specific Boolean strings were developed for each condition. The query structure used was:

(TI=sleep OR AB=sleep OR AK=sleep) AND (PY=XXXX-XXXX) AND (SO="Journal Name”), where:

TI=title, AB=abstract, AK=keywords, PY=year of publication, SO=source title.

For each journal, the total number of publications containing the term “sleep” was recorded for each period. Data were then aggregated by category to determine the absolute sleep-related growth and to calculate the percentage increase between intervals and across the entire 15-year span based on the total publication volume of each category (T1 to T3).

#### Phase II

Journal Output Normalization: to collect the total publication volume for each selected journal across the 3 predefined timeframes and determine the relative contribution of sleep research within each academic category. This normalization procedure allowed for a proportional analysis of sleep-related output independent of the general expansion of scientific publishing. The search was conducted using the following query structure, with placeholders adjusted for each specific journal title and period

(PY=YYYY-YYYY) AND (IS=(XXXX-XXX OR XXXX-XXXX)), where PY=year of publication and IS=ISSN.

Consistent with the initial search criteria, no document type filters were applied to ensure that the total denominator reflected the full editorial activity of each source.

To determine the relative prevalence of sleep research within each academic category, the results were calculated using an aggregate proportional approach. For each timeframe, the total number of sleep-related records (numerator) from the 10 selected journals in a given category was summed and then divided by the combined total publication output (denominator) of those same journals. This process was repeated for each of the 3 timeframes (T1, T2, and T3) to identify longitudinal shifts in the thematic weight of sleep science within each specialized field dividing the number of sleep-related publications/total number of scientific publications.

P=(∑ sleep-related publications/∑ total journal publications) × 100; where P=proportion of sleep-related publications for each category.

#### Phase III

Domain-specific sleep descriptors: to conduct targeted searches for specific sleep variables using descriptors based on Medical Subject Headings (MeSH) adapted for WoS (Table 1). This phase employed the same methodological criteria as the initial screening, including the identical 15-year timeframe (2010–2024) and the same cohort of 60 high-impact journals. To ensure maximum sensitivity and consistency across all disciplines, queries were executed within the WoS Core Collection using the ‘Topic’ (TS) field, which encompasses titles, abstracts and keywords. Specific Boolean strings were developed for each condition. The query structure used was:

IS=(XXXX-XXXX OR XXXX-XXXX) AND PY=(YYYY-YYYY) AND TS=(MeSH terms adapted for WoS), where IS=ISSN, PY=year of publication and TS=topic field.

**Table 1:** Descriptors from MeSH adapted for WoS used in the analysis

Sleep variables	MeSH descriptors
Obstructive sleep apnea	“obstructive sleep apnea” OR “sleep apnea hypopnea syndrome” OR “upper airway resistance sleep apnea syndrome” OR “sleep apnea syndrome” OR OSAHS OR OSA
Insomnia	insomnia OR “chronic insomnia” OR sleeplessness OR “disorders of initiating and maintaining sleep”
Sleep Quality	“sleep quality”
Insufficient sleep	“sleep deprivation” OR “rem sleep deprivation” OR “sleep fragmentation” OR “insufficient sleep” OR “inadequate sleep” OR “sleep debt” OR “sleep insufficiency”
Rapid eye movement sleep behavior disorder	“rem sleep behavior disorder” OR “rapid eye movement sleep behavior disorder” OR “rem behavior disorder”
Sleepwalking	sleepwalking OR “sleep walking” OR “sleep walking disorder” OR “nocturnal wandering”
Periodic limb movement disorder	“periodic limb movement disorder” OR “periodic movement disorder sleep” OR “sleep disorder periodic movements” OR “sleep-related periodic leg movements excessive”
Sleepiness	hypersomnia OR hypersomnolence OR “hypersomnia recurrent” OR “excessive daytime sleepiness” OR “daytime somnolence”
Bruxism	“sleep bruxism” OR “sleep bruxisms” OR “nocturnal bruxism” OR “sleep-related bruxism”

### Data analysis

The data analysis was conducted in 3 phases to evaluate the evolution of sleep research across the selected categories:

1. Absolute Volume Assessment: We first quantified the absolute volume of sleep-related publications within each academic category for each of the 3 defined timeframes (T1, T2, and T3). This stage aimed to identify the growth in scientific output focused on sleep.
2. Proportional and Comparative Analysis: To account for the general expansion of scientific literature, we calculated the thematic share of sleep research. This was determined by the ratio of sleep-related articles to the total publication output of the top 10 journals in each category per period. Relative increase (%) was subsequently calculated between timeframes (T1–T2, T2–T3, and T1–T3) to measure the specific expansion rate of sleep science relative to the overall growth of each discipline.
3. Thematic and Clinical Descriptor Mapping: In the final phase, we conducted an analysis of specific sleep disorders and sleep clinical variables using descriptors from MeSH adapted for WoS search and established descriptors. This stage allowed for the quantification of specific research trends, such as Insomnia, OSA, and REM Sleep Behavior Disorder, providing a qualitative breakdown of how distinct sleep conditions have evolved within the scientific discourse of each category.

## Results

### Screening and inclusion of journals

A total of 60 high-impact journals (top 10 per category of JCR) were identified and included in the analysis (**Supp.** Table 1). Notably, certain journals reached the JCR top-tier ranking despite being established during the analyzed periods (detailed in **Supp.** Table 1). Rather than excluding these sources, they were intentionally retained, with publication counts recorded as zero for the years preceding their inception. This decision was based on the premise that the rapid emergence and ascent of new journals to high-impact status, often becoming dominant voices in their respective fields within a few years, constitutes a significant bibliometric finding. Including these journals provides a more authentic representation of each category’s dynamic growth and the shifting landscape of scientific prestige.

**Fig. 1:**
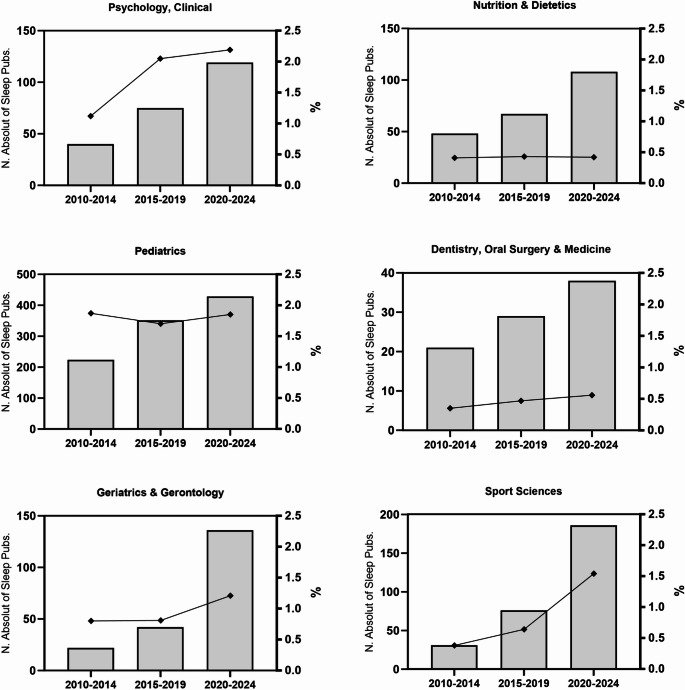
Trends in sleep-related publications across 6 academic categories (2010–2024). Bars (left Y-axis) represent the absolute number of publications containing the term “sleep” in the title, abstract, or keywords. The line graph (right Y-axis) represents the relative proportion (%) of sleep-related research within the total publication volume of the top 10 journals in each field, according to the Journal Citation Reports (JCR). Data were retrieved from the Web of Science Core Collection

**Fig. 2:**
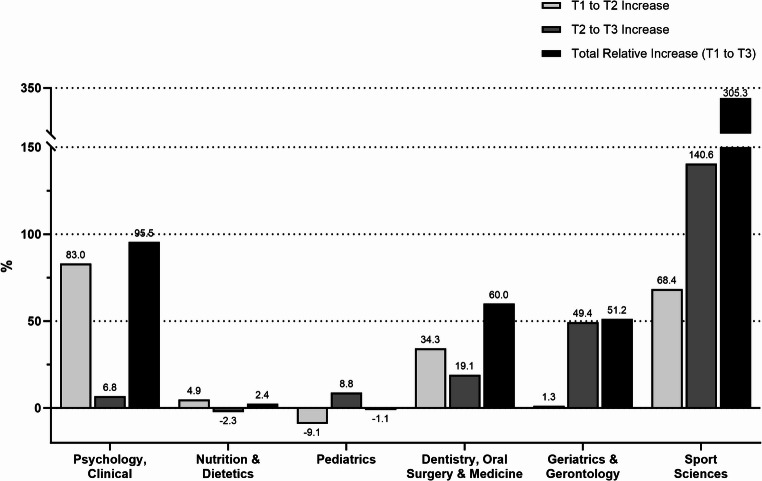
Relative increase or decrease in the proportion of sleep-related research within specialized health categories across 3 time periods (2010–2024)

Through the 15-year study period, Pediatrics emerged as the most prolific field in sleep research, totaling 1,005 publications in sleep, followed by Sports Sciences (*n* = 293), Psychology (*n* = 234), Nutrition (*n* = 223), Geriatrics (*n* = 200) and Dentistry (*n* = 88) (Fig. 1). The analysis of the 3 timeframes revealed a consistent upward trajectory in all journals’ categories. Sport, Psychology, Dentistry and Geriatrics exhibited the most pronounced 15-year period sleep relative growth compared with their overall scientific volume, with 305%, 95.5%, 60.0% and 51.2%, respectively (Fig. 2).

### Journal output normalization

Sleep-related research accounted for 0.4% to 2.2% of the total scientific output across the analyzed categories (Table 2). In most categories, sleep research significantly outpaced the expansion of the overall scientific publications in the field (Table 3). Most notably, in Sport, sleep-related publications grew 10 times than the overall journal volume (500.0% *vs*. 46.9%). Similarly, in Psychology and Dentistry, the growth of sleep research exceeded the general publication increase by 3.7 and 5.4 times, respectively. Pediatrics and Nutrition showed similar increases in sleep-related and overall scientific publications.

**Table 2:** Proportion of sleep-related publications in overall scientific publications of each category analyzed in different timeframes

Journal’s category	T1	T2	T3
Psychology, Clinical	1.1%	2.0%	2.2%
Nutrition & Dietetics	0.4%	0.4%	0.4%
Pediatrics	1.9%	1.7%	1.8%
Dentistry, Oral Surgery & Medicine	0.4%	0.5%	0.6%
Geriatrics & Gerontology	0.8%	0.8%	1.2%
Sport Sciences	0.4%	0.6%	1.5%

Data represents the percentage of sleep-related publications relative to the total scientific output of the top 10 journals in each category per timeframe. Proportions were calculated based on a longitudinal analysis of the Web of Science Core Collection. Data are divided into 3 timeframes: T1 (2010–2014), T2 (2015–2019), and T3 (2020–2024)

**Table 3:** Comparative growth analysis: overall scientific output *vs*. sleep-specific research (2010 to 2024)

Journal’s category	Overall scientific publications increased	Sleep-related publications increase	Difference
Sport Sciences	46.9%	500.0%	10.6 times
Psychology, Clinical	52.4%	197.5%	3.7 times
Dentistry, Oral Surgery	15.0%	81.0%	5.4 times
Geriatrics & Gerontology	310.5%	518.2%	1.6 times
Nutrition & Dietetics	120.2%	125.0%	*Similar*
Pediatrics	93.4%	91.5%	*Similar*

The table displays the cumulative percentage increase in total journal publications compared to the cumulative increase in sleep-related articles from 2010 to 2024. The “Difference” column illustrates the factor by which sleep research expansion outpaced general field growth. “Similar” denotes categories where sleep research growth was proportional to the overall journal expansion

## Prevalence of specific sleep descriptors and disorders

**Fig. 3:**
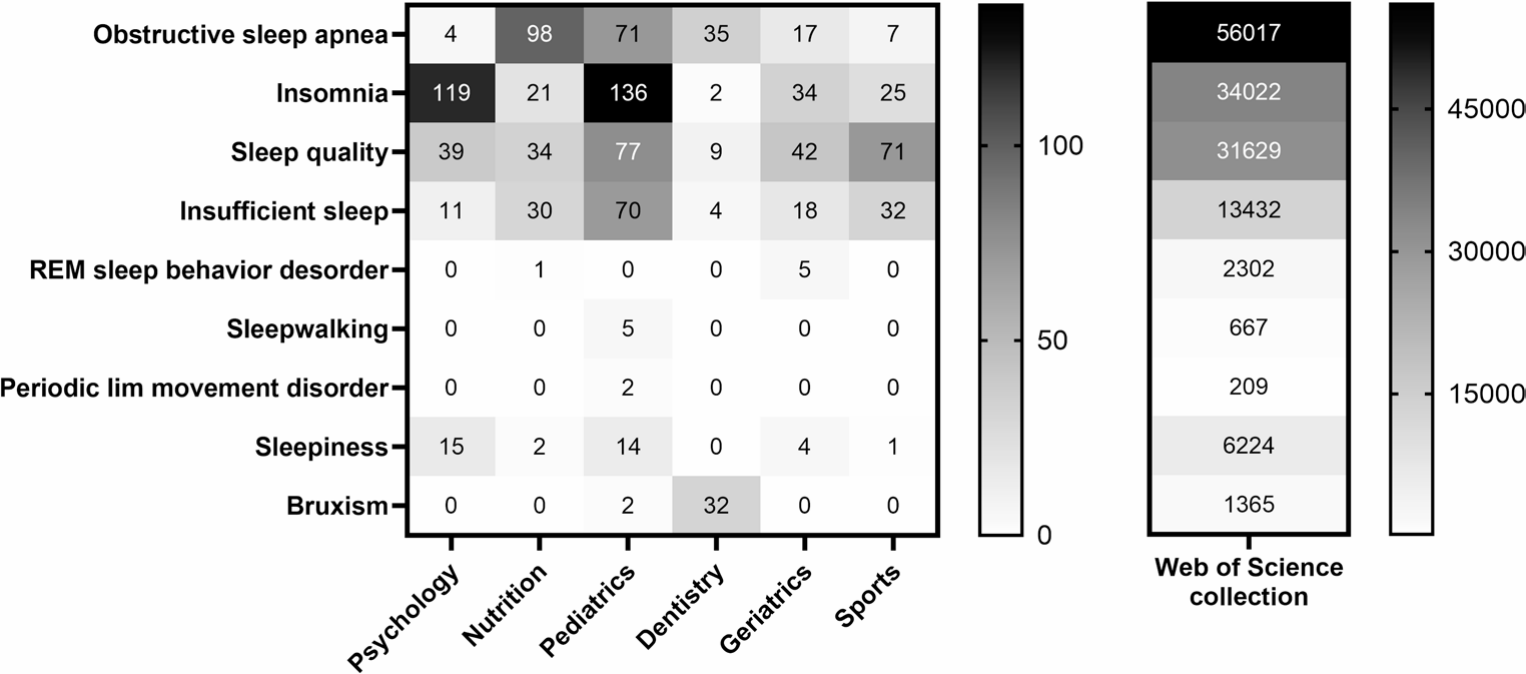
Heatmap of the distribution of specific sleep-related topics within the top 10 scientific journals according to Journal Citation Reports (JCR) across 6 health disciplines

The distribution of sleep-related research themes demonstrated significant variability across the analyzed specialties, although a consistent focus on 3 core variables (insomnia, OSA, and sleep quality) was observed across all categories (Fig. 3). While “sleep quality” emerged as one of the 2 most frequent research topics in 5 of the 6 health domains, dentistry represented a unique thematic outlier in the dataset, as it was the only category to feature both “bruxism” and “OSA” among its top 2-related research priorities.

## Discussion

Our bibliometric analysis underscores a significant paradigm shift, with Sport, Psychology, Dentistry, and Geriatrics emerging as the primary disciplines integrating sleep research into their specialized discourse. While sleep-related research represented a thematic share of 0.4% to 2.2% across all categories, its growth rate was disproportionately higher than general scientific expansion. Notably, in 4 of the 6 analyzed fields, sleep-related output outpaced overall journal growth by more than 1.5-fold. The most striking expansion occurred in Sport, where the growth of sleep research exceeded general category expansion by 10.6 times. Similarly, although Psychology maintained a steadier distribution across timeframes, it achieved a 100% increase in sleep-related output over 15 years, outperforming the field’s general growth by a factor of 3.6. In contrast, Nutrition and Pediatrics demonstrated a more stable trend, with sleep-related publications expanding in strict proportion to the overall increase in scientific output.

The 305.3% surge in sleep-related publications within the Sport Sciences category, a growth rate 10.6 times higher than the field’s general scientific expansion, reflects a paradigm shift in athletic management. This trend aligns with the growing consensus that sleep is not merely a passive state of rest, but an essential, bioactive component of physiological repair and athletic recovery [15]. Clinical evidence underscores this importance, showing that athletes who achieve at least 7 h of sleep daily are twice as likely to resolve injury symptoms compared to their sleep-deprived counterparts [16]. Beyond injury rehabilitation, sleep deprivation is now recognized as a potent disruptor of both physical performance (e.g., strength, speed, and aerobic capacity) and cognitive-psychological resilience, including reaction time and motivational drive [15]. This unprecedented academic interest is likely driven by the heightened awareness of sleep’s influence on competitive outcomes and the widespread integration of wearable technology. The adoption of such devices has become a standardized protocol among professional teams to monitor health parameters and optimize performance through precise sleep-wake tracking.

In contrast to the rapid exponential growth observed in newer fields, the contribution of Psychology remains a foundational and steady pillar of sleep research. Our data show that although Psychology showed similar percentage of sleep-related publication across the 3 timeframes, this category had a 3.7 increase compared to overall scientific expansion. This evidence can be attributed to its long-standing leadership in investigating the behavioral, cognitive, and emotional dimensions of sleep, especially because there is a bidirectional relation between insomnia, the most prevalent sleep disorder, and mental health [10, 11]. The relatively lower percentage increase in this category across the timeframes compared to others, likely reflects a ‘ceiling effect’ of a mature discipline that has already integrated sleep as a core component of mental health research for decades. This sustained productivity underscores Psychology’s role in providing the evidence-based behavioral interventions that now underpin much of the interdisciplinary interest seen in other medical specialties.

Regarding Dentistry, although it accounted for the lowest absolute number of sleep-related records, the category demonstrated a notable 60% increase in thematic output from 2010 to 2024. More importantly, the expansion of sleep research in this field was 5.4 times higher than the overall scientific growth of the analyzed journals. This trend is likely driven by the high prevalence of sleep-disordered breathing [12], which has intensified the demand for specialized dental intervention. It can be confirmed by our results with OSA maintaining the highest publication volume among sleep variables, followed by bruxism in this category. This distribution reinforces the status of OSA as the primary research focal point in dental sleep medicine, while simultaneously highlighting bruxism as a uniquely significant thematic priority within this field. Dentists play an increasingly critical role in identifying oral and anatomical risk factors associated with OSA. Furthermore, recent technological advancements have introduced innovative diagnostic tools and therapeutic options, such as sophisticated mandibular advancement devices and digital imaging, enhancing the precision of both screening and long-term management within dental practice.

While Pediatrics emerged as the most prolific field in our analysis, these findings warrant contextualization. Unlike narrower specialties, Pediatrics serves as a broad umbrella encompassing various subspecialties, such as pediatric pulmonology, neurology, and neonatality, where sleep-related breathing disorders and neurodevelopmental sleep patterns are primary clinical concerns. Therefore, its higher publication volume likely reflects this wide clinical scope rather than a disproportionate dominance over more specialized fields like Dentistry or Sports, which focus on more specific physiological aspects of sleep.

Sleep-related publication was 1.6 times higher than the overall scientific expansion of Geriatrics category reflecting the critical intersection of aging and sleep pathology. As life expectancy increases, the clinical management of age-related sleep fragmentation and non-restorative sleep becomes paramount. In a longitudinal study on aging with 27,210 participants > 45 years, 20% of them presented higher risk of OSA [13], while insomnia exhibits an accumulative pattern across the life span, as observed in a global study by Benjafield et al. [14]. These disorders are not merely symptoms of aging but are drivers of geriatric syndromes, cognitive decline, and cardiovascular morbidity [15–18]. The intensity of recent research in this field suggests that sleep is now viewed as a modifiable risk factor for extending “healthspan” in the elderly.

The Nutrition category demonstrated the highest level of thematic stability, maintaining a consistent proportion of sleep-related publications relative to overall scientific output at approximately 0.4% across all 3 analyzed timeframes. While the total scientific volume within this category expanded by 120.2%, sleep-specific publications grew by 125.0%, indicating that research interest in sleep mirrored the robust growth of the field at large. This steady integration is likely driven by the established multidisciplinary approach to OSA, where nutrition plays a critical role in managing strongly correlated comorbidities such as obesity and metabolic diseases [19]. The fact that OSA was identified as the primary sleep-related term within this category further supports this association. These findings suggest that the metabolic and weight-management aspects of OSA have anchored sleep research within the nutritional sciences, ensuring its proportional representation remains constant despite the overall expansion of the discipline.

Across nearly all analyzed categories, “sleep quality,” “insomnia,” and “OSA” emerged as the predominant research descriptors. The distribution of these terms aligned closely with the clinical focus of each specialty: insomnia was the most frequent descriptor in Psychology and Pediatrics, while OSA predominated in Nutrition and Dentistry. Conversely, sleep quality was the primary focus within the Geriatrics and Sports categories. These findings are consistent with established clinical paradigms. The prevalence of insomnia in psychological and pediatric research reflects its well-documented bidirectional relationship with mental health. The focus on OSA within Nutrition and Dentistry is likely driven by its strong associations with obesity, metabolic conditions, and craniofacial anatomical structures. Furthermore, the emphasis on sleep quality in Geriatrics and Sports categories aligns with the known age-related decline in sleep efficiency and the recognized role of physical activity in improving sleep architecture.

### Limitations

This study has limitations that should be considered when interpreting the results. First, our analysis was restricted to the top 10 JCR journals per field, resulting in a sample of 60 titles. While this selection captures the trends within elite scientific platforms, it does not represent the entirety of the literature in each discipline. Second, the reliance on a single keyword (“sleep”) may have led to the omission of relevant studies that use more specific terminology. Third, this analysis focuses on the quantity of publications and does not account for qualitative metrics or citation-based impact. Finally, the reliance on JCR-indexed journals introduces a potential geographic and language bias, as it may underrepresent regional research or studies published in languages other than English.

## Conclusions

Our findings indicate that diverse health disciplines are increasingly positioning sleep as a relevant variable in human health research. While modern lifestyle challenges often impact sleep hygiene, the scientific community has intensified its focus on the physiological and clinical links between sleep and quality of life. This growing body of literature provides a theoretical foundation that may, in the future, support more integrated and interdisciplinary care models. Moving forward, the continued expansion of sleep research across these domains is expected to further refine our understanding of sleep’s role in systemic health and contribute to the development of more personalized evidence-based approaches within their respective fields.

## Supplementary Information

Below is the link to the electronic supplementary material.

Supplementary Material 1

## Data Availability

The data that support the findings of this study are available on request from the corresponding author, MLA.

## Declarations

### Permission to reproduce material from other sources

Not applicable in our study. This study does not contain reproduced material from other sources.
