# Supplementary material for: The great awakening: A 15-year bibliometric analysis of the global surge in sleep research
Source: Sleep Breath. 2026 Jun 10;30(3):184. doi: 10.1007/s11325-026-03733-9 (PMC13253712; doi:10.1007/s11325-026-03733-9)
Supplement: Supplementary file 1 — Supplementary Material 1 [file 11325_2026_3733_MOESM1_ESM.docx]

**Supp. Table 1**. Detailed publication metrics for individual journals of each category.

| **Psychology, Clinical** | | **FILTERED FOR SLEEP** | | | **TOTAL OF PUBLICATIONS** | | |  | **Nutrition & Dietetics** | | **FILTERED FOR SLEEP** | | | **TOTAL OF PUBLICATIONS** | | |  | **Pediatrics** | | **FILTERED FOR SLEEP** | | | **TOTAL OF PUBLICATIONS** | | |
| --- | --- | --- | --- | --- | --- | --- | --- | --- | --- | --- | --- | --- | --- | --- | --- | --- | --- | --- | --- | --- | --- | --- | --- | --- | --- |
| **Journal** | **ISSN** | **T1** | **T2** | **T3** | **T1** | **T2** | **T3** |  | **Journal** | **ISSN** | **T1** | **T2** | **T3** | **T1** | **T2** | **T3** |  | **Journal** | **ISSN** | **T1** | **T2** | **T3** | **T1** | **T2** | **T3** |
| Annual Review of Clinical Psychology | 1548-5943 1548-5951 | 3 | 2 | 3 | 128 | 105 | 103 |  | Progress in Lipid Research | 0163-7827 1873-2194 | 0 | 0 | 0 | 153 | 133 | 148 |  | JAMA Pediatrics | 2168-6203 2168-6211 | 13 | 34 | 27 | 568 | 1.819 | 1.976 |
| International Journal of Transgender Health | 2689-5269 2689-5277 | 0 | 0 | 1 | 0 | 5 | 351 |  | Annual Review of Nutrition | 0199-9885 1545-4312 | 0 | 2 | 3 | 100 | 103 | 97 |  | *The Lancet Child & Adolescent Health | 2352-4642 2352-4642 | 0 | 5 | 9 | 0 | 600 | 1.058 |
| Clinical Psychology Review | 0272-7358 1873-7811 | 11 | 13 | 7 | 397 | 370 | 403 |  | *Current Obesity Reports | 2162-4968 2162-4968 | 16 | 12 | 11 | 124 | 234 | 221 |  | Journal of the American Academy of Child and Adolescent Psychiatry | 0890-8567 1527-5418 | 5 | 93 | 103 | 996 | 5.570 | 6.932 |
| Health Psychology Review | 1743-7199 1743-7202 | 1 | 7 | 9 | 93 | 185 | 161 |  | Food Chemistry | 0308-8146 1873-7072 | 3 | 1 | 2 | 7.368 | 9330 | 15.593 |  | Pediatrics | 0031-4005 1098-4275 | 126 | 112 | 87 | 4.083 | 5.380 | 5.609 |
| Clinical Psychology-Science and Practice | 0969-5893 1468-2850 | 2 | 0 | 3 | 179 | 196 | 321 |  | Advances in Nutrition | 2161-8313 2156-5376 | 4 | 6 | 14 | 398 | 529 | 781 |  | Pediatric Diabetes | 1399-543X 1399-5448 | 1 | 12 | 18 | 493 | 710 | 582 |
| Clinical Child and Family Psychology Review | 1096-4037 1573-2827 | 0 | 2 | 1 | 110 | 126 | 212 |  | Critical Reviews in Food Science and Nutrition | 1040-8398 1549-7852 | 1 | 6 | 9 | 329 | 1180 | 2.618 |  | Child and Adolescent Mental Health | 1475-357X 1475-3588 | 4 | 3 | 8 | 405 | 277 | 378 |
| Psychological Medicine | 0033-2917 1469-8978 | 16 | 33 | 75 | 1.373 | 1.474 | 2.390 |  | *Hepatobiliary Surgery and Nutrition | 2304-3881 2304-389X | 0 | 1 | 0 | 0 | 523 | 911 |  | European Child & Adolescent Psychiatry | 1018-8827 1435-165X | 34 | 36 | 61 | 1.781 | 1.529 | 1.290 |
| Body Image | 1740-1445 1873-6807 | 0 | 0 | 2 | 370 | 411 | 702 |  | *Food Science and Human Wellness | N/A 2213-4530 | 0 | 1 | 5 | 0 | 100 | 827 |  | Child and Adolescent Psychiatry and Mental Health | N/A 1753-2000 | 1 | 4 | 35 | 72 | 270 | 543 |
| Journal of Consulting and Clinical Psychology | 0022-006X 1939-2117 | 3 | 15 | 10 | 505 | 511 | 404 |  | Clinical Nutrition | 0261-5614 1532-1983 | 4 | 10 | 37 | 808 | 1.464 | 2412 |  | Journal of Adolescent Health | 1054-139X 1879-1972 | 32 | 41 | 70 | 2.300 | 2.779 | 2.855 |
| Child and Adolescent Mental Health | 1475-357X 1475-3588 | 4 | 3 | 8 | 405 | 277 | 378 |  | American Journal of Clinical Nutrition | 0002-9165 1938-3207 | 20 | 28 | 27 | 2.314 | 1.955 | 1.923 |  | Pediatric Critical Care | 1529-7535 1947-3893 | 8 | 12 | 11 | 1.306 | 1.810 | 1.996 |
| **Total** |  | **40** | **75** | **119** | **3560** | **3660** | **5425** |  | **Total** |  | **48** | **67** | **108** | **11594** | **15551** | **25531** |  | **Total** |  | **224** | **352** | **429** | **12004** | **20744** | **23219** |

| **Dentistry, Oral surgery & Medicine** | | **FILTERED FOR SLEEP** | | | **TOTAL OF PUBLICATIONS** | | |  | **Geriatrics & Gerontology** | | **FILTERED FOR SLEEP** | | | **TOTAL OF PUBLICATIONS** | | |  | **Sport Sciences** | | **FILTERED FOR SLEEP** | | | **TOTAL OF PUBLICATIONS** | | |
| --- | --- | --- | --- | --- | --- | --- | --- | --- | --- | --- | --- | --- | --- | --- | --- | --- | --- | --- | --- | --- | --- | --- | --- | --- | --- |
| **Journal** | **ISSN** | **T1** | **T2** | **T3** | **T1** | **T2** | **T3** |  | **Journal** | **ISSN** | **T1** | **T2** | **T3** | **T1** | **T2** | **T3** |  | **Journal** | **ISSN** | **T1** | **T2** | **T3** | **T1** | **T2** | **T3** |
| Periodontology 2000 | 0906-6713 1600-0757 | 0 | 0 | 1 | 196 | 215 | 308 |  | *Nature Aging | N/A 2662-8465 | 0 | 0 | 4 | 0 | 0 | 786 |  | British Journal of Sports Medicine | 0306-3674 1473-0480 | 8 | 17 | 43 | 1.417 | 1.883 | 1.580 |
| International Journal of Oral Science | 1674-2818 2049-3169 | 2 | 1 | 0 | 181 | 162 | 260 |  | *Lancet Healthy Longevity | 2666-7568 2666-7568 | 0 | 0 | 14 | 0 | 0 | 686 |  | *Journal of Sport and Health Science | 2095-2546 2213-2961 | 3 | 5 | 29 | 123 | 378 | 442 |
| Internatiional Endodontic Journal | 0143-2885 1365-2591 | 1 | 1 | 3 | 718 | 815 | 860 |  | Ageing Research Reviews | 1568-1637 1872-9649 | 4 | 7 | 23 | 308 | 410 | 1.239 |  | Sports Medicine | 0112-1642 1179-2035 | 7 | 20 | 33 | 454 | 918 | 961 |
| Journal of Clinical Periodontology | 0303-6979 1600-051X | 1 | 1 | 8 | 759 | 780 | 803 |  | Journal of Cachexia Sarcopenia and Muscle | 2190-5991 2190-6009 | 1 | 2 | 5 | 153 | 469 | 1.148 |  | *Sports Medicine-Open | 2199-1170 2198-9761 | 0 | 6 | 21 | 0 | 224 | 562 |
| Japanese Dental Science Review | 1882-7616 2213-6851 | 2 | 1 | 3 | 101 | 87 | 159 |  | Aging Cell | 1474-9718 1474-9726 | 6 | 4 | 11 | 589 | 754 | 1.288 |  | Journal of Orthopaedic & Sports Physical Therapy | 0190-6011 1938-1344 | 2 | 2 | 7 | 668 | 780 | 470 |
| Dental Materials | 0109-5641 1879-0097 | 0 | 0 | 0 | 886 | 985 | 994 |  | Age and Ageing | 0002-0729 1468-2834 | 8 | 21 | 31 | 1.403 | 2.860 | 3.550 |  | Arthroscopy: The Journal of Arthroscopic and related Surgery | 0749-8063 1526-3231 | 2 | 4 | 12 | 1.426 | 2.438 | 2.477 |
| Journal of Dental Research | 0022-0345 1544-0591 | 7 | 9 | 7 | 1.105 | 1.092 | 928 |  | Aging and Disease | 2152-5250 2152-5250 | 3 | 8 | 17 | 182 | 387 | 795 |  | Biology of Sport | 0860-021X 2083-1862 | 1 | 4 | 19 | 237 | 254 | 474 |
| Journal of Dentistry | 0300-5712 1879-176X | 5 | 12 | 8 | 890 | 850 | 1.556 |  | *npj Aging | N/A 2731-6068 | 0 | 0 | 2 | 0 | 0 | 108 |  | Knee Surgery Sports Traumatology Arthroscopy | 0942-2056 1433-7347 | 2 | 7 | 6 | 1.909 | 2.668 | 2.723 |
| Clinical Oral Implants Research | 0905-7161 1600-0501 | 1 | 1 | 3 | 1.010 | 1.008 | 719 |  | Immunity & Ageing | 1742-4933 1742-4933 | 0 | 0 | 0 | 108 | 142 | 318 |  | Exercise and Sport Sciences Reviews | 0091-6331 1538-3008 | 0 | 1 | 3 | 179 | 173 | 141 |
| Progress in Orthodontics | 2196-1042 2196-1042 | 2 | 3 | 5 | 98 | 222 | 246 |  | GeroScience | 2509-2715 2509-2723 | 0 | 0 | 29 | 0 | 177 | 1.343 |  | American Journal of Sports Medicine | 0363-5465 1552-3365 | 6 | 10 | 13 | 1.828 | 2.215 | 2.273 |
| **Total** |  | **21** | **29** | **38** | **5944** | **6216** | **6833** |  | **Total** |  | **22** | **42** | **136** | **2743** | **5199** | **11261** |  | **Total** |  | **31** | **76** | **186** | **8241** | **11931** | **12103** |

The table presents the absolute number of sleep-related records ("Filtered for Sleep") and the total scientific output for each of the top 10 journals per category. Data are divided into 3 timeframes: T1 (2010–2014), T2 (2015–2019), and T3 (2020–2024). ISSN= International Standard Serial Number.

Asterisks (*) denote journals established or indexed during the analyzed period. ISSN and e-ISSN are provided for each source title.
